# *In vivo* quantification and perturbation of Myc-Max interactions and the impact on oncogenic potential

**DOI:** 10.18632/oncotarget.2588

**Published:** 2014-10-12

**Authors:** Philipp Raffeiner, Ruth Röck, Andrea Schraffl, Markus Hartl, Jonathan R. Hart, Kim D. Janda, Peter K. Vogt, Eduard Stefan, Klaus Bister

**Affiliations:** ^1^ Institute of Biochemistry and Center for Molecular Biosciences, University of Innsbruck, Innsbruck, Austria; ^2^ Department of Molecular and Experimental Medicine, The Scripps Research Institute, La Jolla, CA; ^3^ Department of Chemistry, The Scripps Research Institute, La Jolla, CA

**Keywords:** transcription factor, protein-protein interactions, biosensor, small-molecule inhibitors, cancer

## Abstract

The oncogenic bHLH-LZ transcription factor Myc forms binary complexes with its binding partner Max. These and other bHLH-LZ-based protein-protein interactions (PPI) in the Myc-Max network are essential for the physiological and oncogenic activities of Myc. We have generated a genetically determined and highly specific protein-fragment complementation assay based on *Renilla* luciferase to analyze the dynamic interplay of bHLH-LZ transcription factors Myc, Max, and Mxd1 *in vivo*. We also applied this PPI reporter to quantify alterations of nuclear Myc-Max complexes in response to mutational events, competitive binding by the transcriptional repressor Mxd1, or perturbations by small-molecule Myc inhibitors, including recently identified potent PPI inhibitors from a Kröhnke pyridine library. We show that the specificity of Myc-Max PPI reduction by the pyridine inhibitors directly correlates with their efficient and highly specific potential to interfere with the proliferation of human and avian tumor cells displaying deregulated Myc expression. In a direct comparison with known Myc inhibitors using human and avian cell systems, the pyridine compounds reveal a unique inhibitory potential even at sub-micromolar concentrations combined with remarkable specificity for the inhibition of Myc-driven tumor cell proliferation. Furthermore, we show in direct comparisons using defined avian cell systems that different Max PPI profiles for the variant members of the Myc protein family (c-Myc, v-Myc, N-Myc, L-Myc) correlate with their diverse oncogenic potential and their variable sensitivity to the novel pyridine inhibitors.

## INTRODUCTION

The *myc* oncogene was originally discovered as the transforming principle (v-*myc*) in the genome of avian acute leukemia virus MC29 [1]. The v-*myc* allele is derived from the chicken cellular protooncogene c-*myc* by retroviral transduction [2, 3]. The Myc protein product, initially identified as a viral Gag-Myc hybrid protein encoded by MC29 genomic RNA [4], is a transcriptional regulator of the basic/helix-loop-helix/leucine zipper (bHLH-LZ) protein family, forms heterodimers with the bHLH-LZ protein Max, binds to specific DNA sequence elements (E-boxes, preferentially CACGTG), and is the central node of a universal transcription factor network [5-8]. In human cells, Myc transcription factor circuits control thousands of genes involved in essential cellular processes like growth, proliferation, differentiation, biosynthesis, energy metabolism, and apoptosis [7, 8]. Recent studies suggest that Myc, rather than acting as a conventional transcription factor with a specific set of target genes, can function as a general amplifier of transcription [9, 10]. The principal biochemical activity of the Myc-Max complex is transcriptional activation, but Myc can also act as a transcriptional repressor of specific target genes [7, 8, 11]. The discovery of chromosomal rearrangements of the human *MYC* gene in Burkitt's lymphoma was the first link of the cellular homolog of a retroviral oncogene to human cancer [12]. Today deregulated *MYC* expression is recognized as a crucial driving force in many, if not most human cancers [2, 7, 8]. Mutations in the coding region of *MYC* are not required for oncogenicity and do not play important roles in human cancer, but can enhance the oncogenic potential like in v-*myc* [2].

Because of its pivotal role in cancer, Myc has become an obvious target for attempting to identify small molecule inhibitors with therapeutic potential. However, Myc is not structured like an enzyme with a well defined catalytic cleft; it is even intrinsically disordered in free form [13], and all its biochemical and biological activities are based on macromolecular interactions, in particular protein-protein interactions (PPIs) involving large flat surface areas that are difficult to target with small molecules [14]. Nevertheless, experimental inhibition of Myc functions in cultured cells has been achieved by small molecules interfering with Myc-Max dimerization [15-17]. Recently, efficient and specific inhibitors of Myc-Max dimerization were identified that are also capable to halt Myc-driven tumor growth *in vivo* [18]. We have described a genetically determined and highly specific protein-fragment complementation assay (PCA) based on the *Renilla* luciferase (*R*luc) and have applied it to a quantitative analysis of Myc-Max interaction in living cells [18]. Here, we use this reporter assay to more broadly characterize PPIs of the Myc-Max network. We also compare the effects of different Myc inhibitors on Myc-Max dimerization and on human and avian Myc-driven cell transformation and proliferation. We document the different oncogenic potentials of the c-Myc, v-Myc, N-Myc, and L-Myc proteins and correlate these activities with the ability to interact with Max and with the sensitivity to specific inhibitors.

## RESULTS

### Quantitative analysis of Myc-Max dimerization in cells

**Fig.1:**
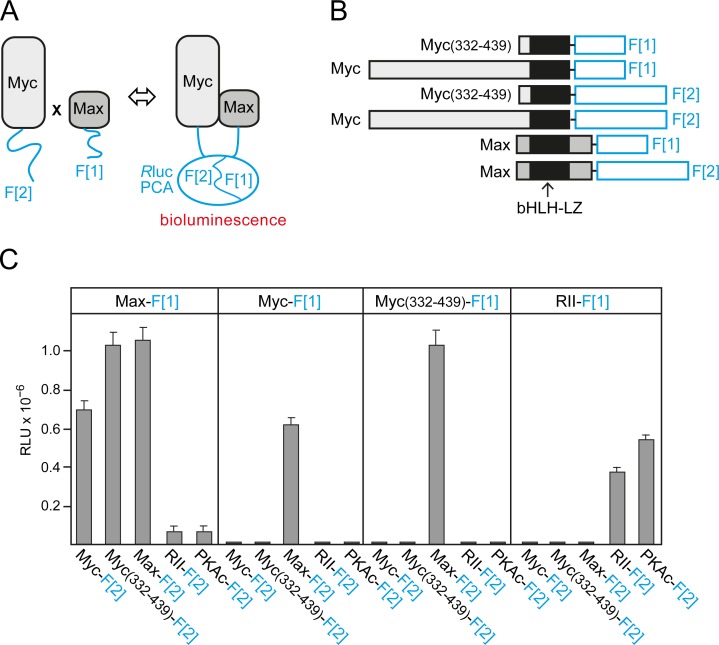
*Rluc*-PCA design and quantification of cellular Myc-Max complexes *(A)* Schematic depiction of the *R*luc-PCA based PPI reporter for the *in vivo* quantification of complex formation of Max and Myc proteins fused to the *R*luc-PCA fragments 1 (F[1]) and 2 (F[2]), respectively. *(B)* The bHLH-LZ transcription factors Max (full length, aa 1-160) and Myc (full length, aa 1-439; or C-terminal fragment, aa 332-439) were fused at the C terminus to an interjacent 10-aa linker (GGGGS)_2_ and the *R*luc-PCA fragments F[1] or F[2]. *(C) R*luc-PCA signals were detected from resuspended HEK293 cells, transiently transfected with the indicated *R*luc-PCA pairs and aliquoted to 96-well white-walled microtiter plates (representative experiment of n=3; ± SD of triplicates; RLU, relative light units). *R*luc-PCA constructs based on PKA subunits (RII, PKAc) were used as controls.

Binary PPIs of Myc, Max, and Mxd1 control cell growth, proliferation, survival, and differentiation in different ways [6-8]. We recently adapted a PCA based on the *R*luc [19, 20] to quantify and dissect complex formation of bHLH-LZ transcription factor dimers directly in the living cell [18]. The general principle of the *R*luc-PCA is based on PPI-dependent folding and complementation of the luciferase reporter when two proteins fused to *R*luc fragment 1 (F[1]) and to fragment 2 (F[2]) interact (Fig. 1*A*). The highly specific *R*luc-PCA for Myc-Max PPI detection [18] was used to quantify the effect of various factors on Myc-Max complex formation, the most important PPI for Myc-driven cancer cell proliferation. We fused complementary fragments of the previously described *R*luc-PCA to the carboxyl (C) terminus (amino acids 332-439) of human c-Myc or to full-length human Myc and to full-length chicken Max (Fig. 1*B*). Human and chicken Max show 100% amino acid identity in the bHLH-LZ region (Fig. S1*A*). A 10-aa encoding linker was inserted between the PCA fragments and the transcription factor [20]. Combinations of the indicated *R*luc-PCA constructs including the control protein kinase A (PKA) reporter [20, 21] were transiently overexpressed in HEK293 cells. Using the *R*luc-PCA signal generated by the addition of the luciferase substrate benzyl-coelenterazine as a read out, we observed complex formation of homo- and heterodimers of the control PKA reporter, as expected (Fig. 1*C*). We also observed strong signals indicating heterodimeric complexes of the *R*luc-PCA-tagged transcription factor pairs Myc-Max and Myc(332-439)-Max. Myc does not form homodimers under physiological conditions [8, 13, 22], and we did not observe signals from PCA pairs containing Myc fragments only. However, we detected strong signals indicating Max-Max homodimer formation (Fig.1*C*) in agreement with published data [23]. Notably, PCA reporter constructs for Myc and Max were only functional when the fragments were in parallel orientation (Fig. 1*B*) as required for bHLH-LZ interactions [22, 23]. As a control, we did not detect significant luminescence signals of mixed pairs of PKA and Myc/Max PCA reporters (Fig. 1*C*). Protein expression of all PCA reporter constructs used was monitored by immunoblotting (Fig. S1*B*). Transcription factor complexes of the Myc-Max network perform their key functions as homo- and heterodimers in the nucleus [6, 8]. Localization of *R*luc-PCA complexes using *R*luc signals as a read-out is technically challenging and provides images with less resolution than a fluorescence-based reporter. To confirm that PCA-tagged Myc-Max complexes are correctly localized in cells, we replaced *R*luc-PCA fragments with Venus-YFP PCA fragments [24, 25] to obtain Venus-YFP-based PCA reporters for Myc-Max complexes (Fig. S2*A*,*B*). In agreement with previous visualization studies on truncated Myc fragments [26], we observed nuclear complexes of full-length Myc-Max, C-terminal Myc(332-439)-Max, and Max-Max (Fig. S2*C*). As a control, the Venus-YFP PCA reporter for PKA regulatory type II subunit (RII) homodimers showed cytoplasmic fluorescence (Fig. S2*C*).

**Fig.2:**
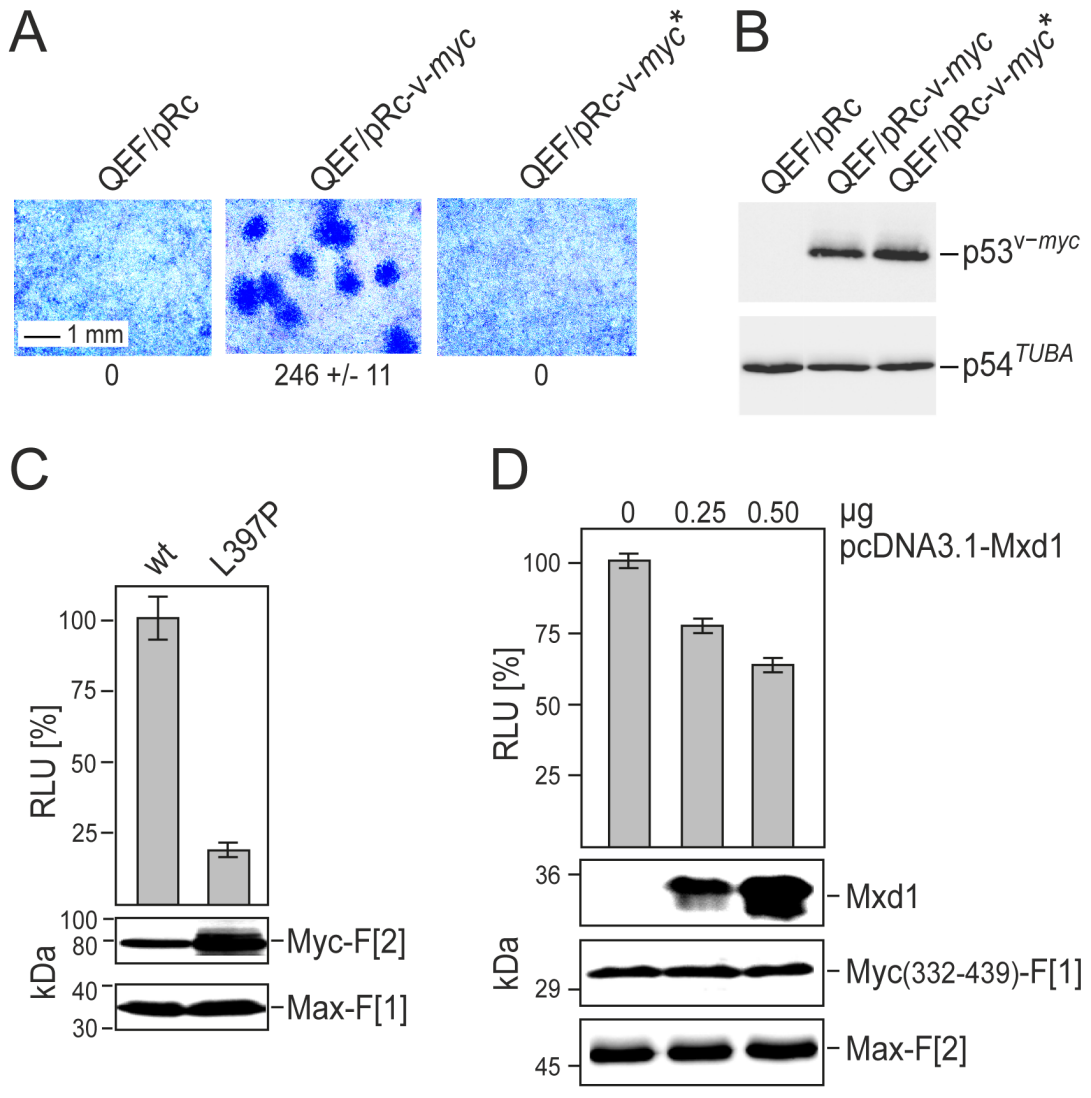
Correlation of Myc cell-transforming potential and Myc-Max interaction *(A)* Cell transforming potential of v-Myc and the dimerization-defective mutant v-Myc* (L397P). Quail embryo fibroblasts (QEF) were transfected with 6-μg aliquots of the plasmids pRc-*v-myc*, pRc-v-myc*, or with the empty pRc vector. Cells were kept under agar overlay for two weeks and then stained with eosin methylene blue. Foci were counted on 60-mm dishes (a section is shown; representative experiment of n=2, ± SD of triplicates). *(B)* Overexpressed HA-tagged v-Myc or v-Myc* proteins, and endogenous tubulin were analyzed by immunoblot analyses of QEF cell extracts prepared one day after transfection. *(C)* PPI of Myc-Max were quantified in *R*luc-PCA experiments. *R*luc-PCA signals were detected from chemically transformed QT6 cells transiently expressing Max-F[1]:v-Myc-F[2] (wt) or Max-F[1]:v-Myc*-F[2] (L397P) PCA pairs. *R*luc-PCA tagged hybrid proteins were analyzed by immunoblot analysis. *(D)* SW480 cells stably expressing the *R*Luc-PCA pair Myc(332-439)-F[1]:Max-F[2] were subjected to bioluminescence analysis following transient expression of Mxd1 (HA-tagged). Increasing amounts of pcDNA3.1-Mxd1 vector DNA were transfected (representative of n=3; ± SD from triplicates). Expression of the PCA hybrid proteins and of Mxd1 was analyzed by immunoblotting.

Mutations of the LZ-motif can reduce Myc-Max dimerization and Myc function *in vitro* and *in vivo* [5, 27]. We confirmed that the L397P mutation of v-Myc (corresponding to L420P in human c-Myc) completely abolished its oncogenic potential as assayed by focus formation of transfected primary quail embryo fibroblasts (QEF) (Fig. 2*A*). Equal expression levels of wild type v-Myc and mutated v-Myc(L397P) in transfected QEF were confirmed by immunoblotting (Fig. 2*B*). Comparing *R*luc-PCA Myc-Max reporter constructs based on wild type v-Myc with mutant v-Myc, we observed an approximately 80% reduction of dimerization for the mutant despite significantly elevated expression levels (Fig. 2*C*). Myc-Max complex formation and Myc function are also affected by competing bHLH-LZ transcription factor interactions. We demonstrated this effect with the Mxd1 (or Mad1) protein which dimerizes with Max [6, 8, 28]. We generated SW480 colorectal adenocarcinoma cells stably transfected with the Myc(332-439)-Max *R*luc-PCA. Transient overexpression of Mxd1 in these cells competed for Max binding and caused a dose-dependent reduction of PCA signals (Fig. 2*D*). Overexpression of Mxd1 had no effect on the control *R*luc-PCA measuring PKA RII subunit dimerization (Fig. S3*A*). Posttranslational modifications also modulate Myc function. Phosphorylation sites of Myc affect both stability and PPIs [29, 30]. Residues S373 and T400 in the human c-Myc HLH region can be phosphorylated by p21-activated kinase, leading to disturbance of Myc-Max interaction [29]. We substituted glutamic acid for these residues to mimic phosphorylation, and used these mutants for PCA analysis. The double mutation of T400E and S373E in c-Myc led to a reduction of Myc-Max complex formation by approximately 40% compared to the wild type control (Fig. S3*B*). Collectively, our data on the LZ mutation, Mxd1 competition, and phosphorylation site mutations demonstrate the specificity, sensitivity, and reliability of the *R*luc-PCA as a tool to monitor PPI in the Myc-Max network.

### Effect of Myc inhibitors on Myc-Max complex formation and human cancer cell proliferation

**Fig.3:**
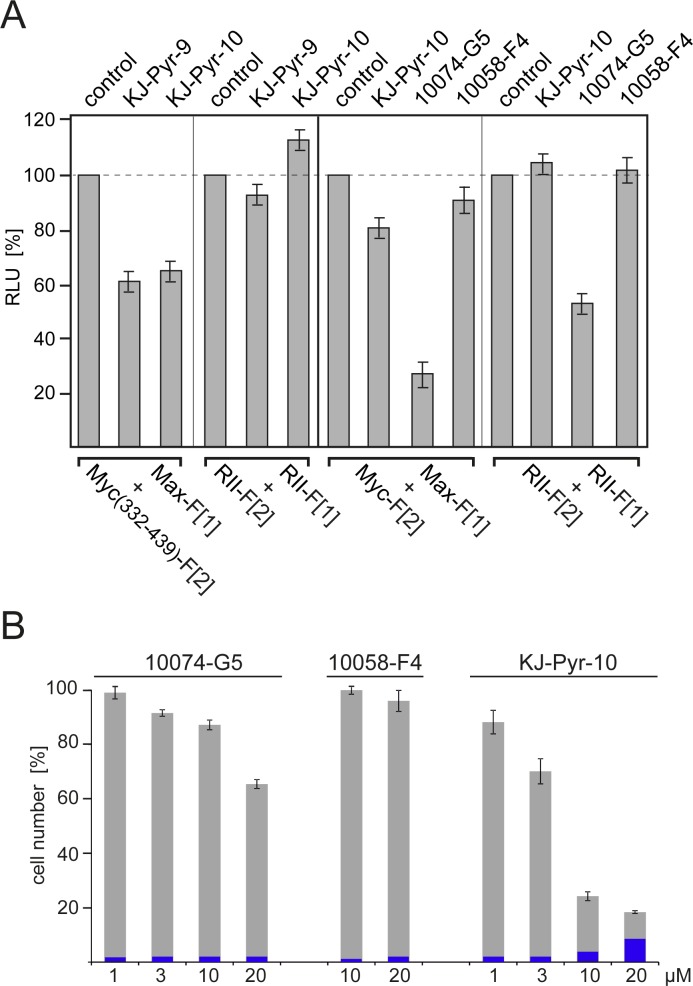
Effect of small-molecule Myc inhibitors on cellular PPI and cell proliferation *(A)* HEK293 cells were transiently transfected with the indicated *R*luc-PCA Myc:Max (cf. Fig. 1*B*) or PKA-based RII:RII expression constructs. Bioluminescence was detected from control cells, or cells treated for 6 h with KJ-Pyr-9, KJ-Pyr-10, 10074-G5, or 10058-F4 (all at 20 μM) (± SEM from at least n=4 independent experiments). *(B)* Effect of Myc inhibitors on human cancer cell proliferation. Dose-dependent effects of the indicated Myc inhibitors on proliferation of the human T-cell leukemia cell line MOLT-4. Cells were exposed to the compounds for 72 h. Cell counts in percent of untreated control cell numbers were determined, and the fraction of non-viable cells (shown in blue) was quantified using a trypan blue assay (± SD of n=3 independent experiments).

Disruption of the Myc-Max PPI leads to a reduction of DNA binding, of transcriptional activity and of oncogenic potential [15-18]. Here we used the PCA reporters to compare the effects of the established Myc inhibitors 10074-G5 and 10058-F4 [16, 17] with the recently published KJ-Pyr-9 and KJ-Pyr-10 compounds [18]. The structures of all inhibitors are shown in Fig. S4. KJ-Pyr-9 and KJ-Pyr-10 reduced complex formation of Myc(332-439):Max by approximately 35-40% (Fig. 3*A*) in agreement with published results [18]. Both compounds had little or no effect on homodimerization of the RII subunits of PKA used as control. We also compared the effect of KJ-Pyr-10, 10074-G5, and 10058-F4 on complex formation of full-length Myc with Max using the *R*luc-PCA Myc-Max biosensor. Since 10074-G5 showed a nonspecific reduction of the PKA control signal, we standardized the reduction rates for the Myc-Max complex formation relative to this control and obtained inhibition of 24% for KJ-Pyr-10, 26% for 10074-G5, and 11% for 10058-F4 (Fig. 3*A*). In order to compare the properties of these inhibitors in the *R*luc-PCA with their efficacy and specificity in inhibition of Myc-driven cell proliferation, we tested the dose- and time-dependent effects of KJ-Pyr-10, 10074-G5, and 10058-F4 on the proliferation of the human lymphoblastic leukemia cell line MOLT-4 which displays high Myc expression levels [31]. KJ-Pyr-10 showed the strongest dose-dependent inhibition of MOLT-4 cell proliferation (Fig. 3*B*) and a one-time application of KJ-Pyr-10 for 72 h at 1 μM was sufficient to inhibit proliferation of MOLT-4 cells. Concentrations of KJ-Pyr-10 beyond 10 μM led to increased cell death (Fig. 3*B*).

### Specificity of Myc inhibitors

**Fig.4:**
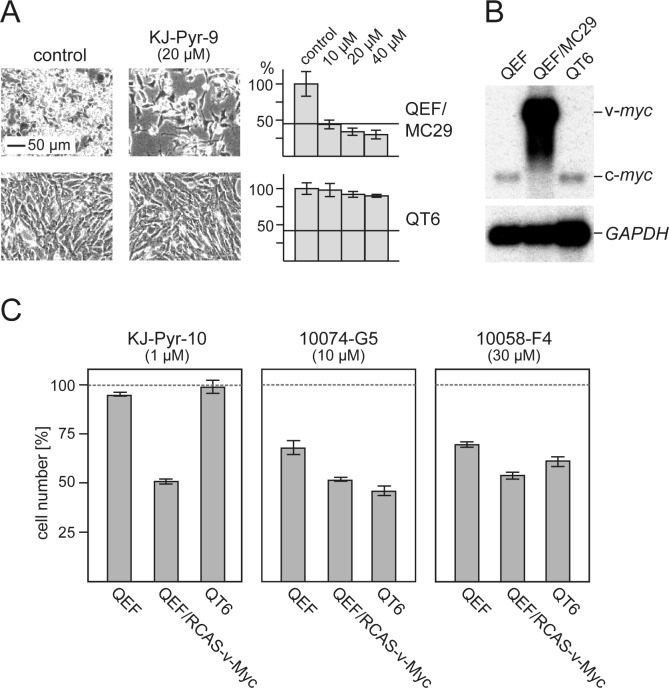
Efficacy and specificity of small-molecule Myc inhibitors *(A)* Cells from the v-*myc*-transformed quail cell line QEF/MC29 or the methylcholanthrene-transformed quail cell line QT6 were treated for 24 h with inhibitor compound KJ-Pyr-9 at the indicated concentrations. Then, cells were counted and microphotographs were taken. Average numbers of control cells were set to 100%. Horizontal bars indicate the numbers of cells initially seeded. *(B)* Northern analysis of RNAs from QEF/MC29, QT6, and normal QEF using a *myc*-specific probe. The positions of v-*myc* (MC29 genomic) and c-*myc* mRNAs are indicated. Hybridization with a *GAPDH*-specific probe was used as RNA loading control. *(C)* Normal QEF, QEF transformed with an RCAS-v-Myc construct, and QT6 cells were treated for 48 h with inhibitor compounds KJ-Pyr-10, 10074-G5, or 10058-F4 at the indicated concentrations. Cell counts were determined and the numbers of control cells were set to 100% (representative of n=3, ± SD from triplicates).

We used avian cells to compare KJ-Pyr-9 and KJ-Pyr-10 with 10074-G5 and 10058-F4 in greater detail. Three cell types were selected for this study: normal QEF, Myc-driven QEF (transformed by the MC29 retrovirus), and QT6, a continuous quail cell line derived from a methylcholanthrene-induced fibrosarcoma [32]. The KJ-Pyr-9 inhibitor specifically inhibited the proliferation of the Myc-driven, MC29-transformed QEF, but it had virtually no effect on the chemically transformed QT6, even at the highest dose tested (Fig. 4*A*). Notably, Northern blot analysis confirmed that QT6 cells show normal c-*myc* expression, and QEF/MC29 display high v-*myc* expression in form of MC29 genomic RNA (Fig. 4*B*). In a similar comparison, we used again normal QEF and QT6, but for the Myc-driven QEF, we replaced MC29 with the RCAS-v-Myc vector that expresses v-Myc without the viral gag sequences. KJ-Pyr-9, KJ-Pyr-10 specifically inhibited the proliferation of Myc-driven cells by 50% at 1 μM, but did not affect normal QEF and QT6 (Fig. 4*C*). 10074-G5 and 10058-F4 reached similar levels of inhibition at higher concentrations (10 μM and 30 μM respectively), but at those concentrations also affected the replication of normal and chemically transformed cells (Fig. 4*C*). We also compared the effects of KJ-Pyr-10, 10074-G5, and 10058-F4 on the proliferation of the tet-off quail cell line Q/tM8 conditionally transformed by a doxycycline controlled *v-myc* oncogene [33]. KJ-Pyr-10 and 10074-G5 showed the strongest inhibitory effect, particularly when the cells were subjected to a dox-/dox+/dox-cycle to induce reversion and re-initiation of the transformed phenotype (Fig. S5). Again, the pyridine inhibitor showed the strongest effect at lower concentrations. In summary, the comparative analyses confirmed the potency and specificity of the pyridine inhibitors KJ-Pyr-9 and KJ-Pyr-10.

### Comparative analysis of oncogenicity, PPI, and inhibition of Myc family members

**Fig.5:**
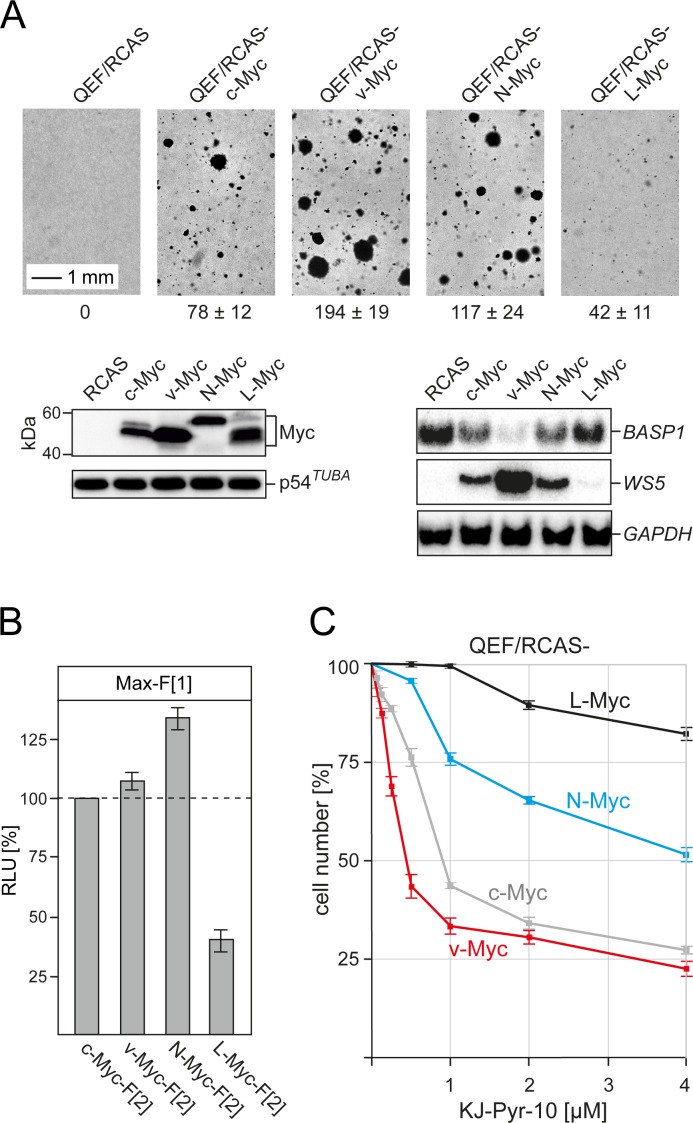
Oncogenicity, PPI quantification, and inhibition of chicken Myc family members *(A)* Cell transformation of primary QEF transfected with retroviral expression vectors (RCAS) carrying the Myc coding regions of chicken c-*myc*, N-*myc*, and L-*myc*, or MC29 v-*myc* was determined in a colony assay. Numbers of colonies formed after 17 d per 1,000 cells seeded are shown below the bright-field micrographs (representative of n=3 independent experiments; ± SD from triplicates). Expression of the HA-tagged Myc family proteins was analyzed by immunoblotting using anti-HA antibodies (*left panels*). An anti-α-tubulin antibody was used for control. The expression of target genes suppressed (*BASP1*) or activated (*WS5*) by v-Myc [35, 36] was monitored by Northern blotting using gene-specific probes (*right panels*). Hybridization with a *GAPDH* probe was used as loading control. *(B)* PPI of Myc variants with Max was determined in *R*luc-PCA experiments of transiently transfected QT6 cells. Bioluminescence signals from four independent experiments were normalized to the expression levels of the Myc variant proteins determined by immunoblotting and densitometric quantification (± SD). *(C)* Dose-dependent effect of inhibitor KJ-Pyr-10 on the proliferation of QEF cells transformed with the various chicken Myc family proteins. In each case, cell counts were determined after 72-h exposure to the inhibitor and are shown in percent of the numbers of untreated control cells (± SD from n=3 independent experiments).

In addition to c-Myc, the human Myc transcription factor family includes the paralogs N-Myc and L-Myc with different oncogenic potential and tumor specificity [2, 8, 34]. We have cloned the coding regions of the chicken orthologs of N-Myc and L-Myc genes (Fig. S6) into the retroviral RCAS vector to directly compare the oncogenic potential of c-Myc, v-Myc, N-Myc, and L-Myc in QEF. In an assay for anchorage-independent growth, v-Myc showed the highest potential to induce colony formation in nutrient agar, followed by N-Myc and c-Myc (Fig. 5*A*). Expression of L-Myc induced only low numbers of small colonies. We also analyzed the effect of the four Myc variants on the expression of two previously characterized transcriptional targets of v-Myc, the activated *WS5* gene [35] and the suppressed *BASP1* gene [36]. Strikingly, the extent of expression modulation of these transcriptional targets correlated exactly with the oncogenic potential as determined in the colony assay (Fig. 5*A*). We also quantified the potential of the four Myc variants to dimerize with Max by using the *R*luc-PCA. Following transient transfections of equal amounts of expression vectors into QT6 cells and normalization on Myc protein expression levels, we observed strong interactions of N-Myc, v-Myc, and c-Myc (in decreasing order) with Max, but substantially lower PPI signals for the L-Myc-Max interaction (Fig. 5*B*). This quantification of PPIs with Max correlates with the oncogenic potential of the four proteins in the avian cell system.

In order to comparatively analyze the effect of pyridine-based inhibitors on the four Myc variants, we established QEF cell lines transformed by the RCAS constructs specifying c-Myc, v-Myc, N-Myc, and L-Myc. After 25 passages, all transformed cells showed enhanced doubling times [v-Myc (21 h ±1), N-Myc (22 h ±1), c-Myc (23 h ±1), L-Myc (27 h ±1)] in comparison to normal QEF (49 h ±4). The effect of inhibitor KJ-Pyr-10 on the proliferation of these cells was analyzed in a dose-dependent manner (Fig. 5*C*). The proliferation of v-Myc- and c-Myc-transformed cells suffered the strongest inhibition, followed by N-Myc-transformed cells. In contrast, the proliferation of L-Myc-transformed cells was only moderately inhibited, even at the highest inhibitor dose. Strikingly, the proliferation of cells driven by the most strongly oncogenic Myc variant, v-Myc, was substantially inhibited by KJ-Pyr-10 at nanomolar concentrations (Fig. 5*C*).

## DISCUSSION

Deregulation of Myc expression is a striking hallmark of many human tumors [2, 7, 8, 34, 37]. In view of the remarkable success in the development of small-molecule inhibitors for the therapy of cancers that are driven by oncogenes encoding kinases [38, 39], Myc also became a compelling target for possible drug development. However, Myc has no enzymatic function, and the obvious target for inhibition with small molecules - the interaction with Max - is difficult to disrupt effectively. PPIs are challenging targets for small-molecule inhibitors [14, 40], although there are examples of PPI inhibitors in the clinic [41-43]. In spite of these principal difficulties, experimental inhibition of Myc-Max dimerization with small molecules has made steady progress [15-18]. In the present study, we have used a novel PCA tool to quantify PPIs in the Myc-Max network, and have also compared several inhibitors of Myc-Max dimerization. The results demonstrate that the Myc-Max *R*luc-PCA is a specific and sensitive reporter to monitor PPI in the Myc-Max network. It reliably registers perturbations of Myc-Max interactions caused by single amino acid substitutions known to negatively regulate Myc function. It also dynamically registers perturbation of the Myc-Max PPI by competing bHLH-LZ proteins like Mxd1, or the inhibition of Myc-Max complex formation by small-molecule inhibitors. Notably, the PPI signals of the Myc-Max *R*luc-PCA are generated in living cells, and the signals from an analogous fluorescent reporter indicate the correct cellular compartimentalization of Myc-Max PPI. Based on these properties, the Myc-Max PCA could be a useful screening tool, also for monitoring chemical optimization of isolated small-molecule inhibitors.

Although there is some evidence for Max-independent activities of Myc, the principal biochemical, biological, and oncogenic functions of Myc family proteins are dependent on the dimerization with Max [6, 8, 11]. Hence, the preferred strategy for the development of possible Myc inhibitors has been the search for small molecules that can interfere with Myc-Max PPI [15-17, 44]. Some of the original isolates were useful tools for experimental inhibition of Myc-Max interaction and Myc-induced transformation of cultured cells, but were not successfully applied *in vivo* due to the lack of adequate pharmacokinetic properties. For two of these compounds, 10058-F4 and 10074-G5, specific binding sites on Myc and inhibitory mechanisms have been proposed [17, 44]. Both molecules stabilize the Myc monomer which is intrinsically disordered and assumes its defined structure only after binding to Max [13, 22]. The recently identified inhibitors, KJ-Pyr-9 and KJ-Pyr-10, were shown to be highly effective and specific both for the inhibition of the Myc-Max complex formation and of Myc oncogenicity as well as for Myc-mediated transcription. KJ-Pyr-9 was also tested *in vivo* and shown to halt the growth of a xenograft of human breast cancer cells [18]. We have now extended the characterization of KJ-Pyr-9 and KJ-Pyr-10 and compared them with 10058-F4 and 10074-G5. The results (Figs. 3, 4 and S5) confirm and extend the efficacy and specificity of KJ-Pyr-9 and KJ-Pyr-10 in inhibiting Myc-Max dimerization and the growth of Myc-driven human and avian cells. The pyridine inhibitors are effective at nanomolar concentrations against v-*myc*-transformed quail cells, but show no effect on normal or chemically transformed QT6 cells. Yet QT6 cells are as vigorously proliferating as QEF/MC29; the critical difference being the level of Myc expression which is much higher in the latter (Fig. 4). These data document the specificity of the pyridine inhibitors for Myc-driven cells.

The avian cell system has the advantage to allow for quantification of transforming events induced by single oncogenes in cultured primary cells. We used this approach to directly compare all members of the chicken Myc family (c-Myc, N-Myc, L-Myc, and c-Myc-derived v-Myc) with respect to their oncogenic potential, target gene regulation, affinity for dimerization with Max, and their sensitivity to KJ-Pyr-10. The oncogenic potential of the four proteins directly correlates with their effect on the expression of v-Myc target genes and with the dimerization signal detected by PCA. Our results on the relative oncogenic potency of Myc proteins are also in agreement with comparable studies measuring transformation of murine fibroblasts cotransfected with *ras* [34]. In view of the broad significance of c-Myc in the etiology of human cancers and the impact of N-Myc and L-Myc in specific tumors like neuroblastoma and small cell lung cancer, the effectiveness of the pyridine inhibitors justifies further efforts to improve the pharmacokinetic and solubility properties. Binding sites for KJ-Pyr-9 and KJ-Pyr-10 need to be identified, and the molecular mechanisms of Myc inhibition need to be determined.

## MATERIALS AND METHODS

### Cell culture

Human embryonic kidney cells (HEK293) and the colon adenocarcinoma cell line (SW480) were grown in DMEM supplemented with 10% (vol/vol) FBS. The human T-cell leukemia cell line (MOLT-4) was grown in RPMI medium supplemented with 10% (vol/vol) FBS. DNA transfection was carried out using TransFectin (Biorad). To generate stably transfected cells expressing *R*Luc-PCA pairs, SW480 cells were co-transfected with *R*Luc-PCA pcDNA3.1 constructs containing hygromycin or zeocin resistance genes. Selection was performed by adding 350 μg/ml of each antibiotic to the cell culture medium. Normal quail embryo fibroblasts (QEF) were prepared from 9-day-old embryos of *Coturnix japonica* as described [4, 35]. The quail cell line QT6 transformed by the chemical carcinogen methylcholanthrene and the cell line Q/tM8 conditionally transformed by v-*myc* have been described before [32, 33]. DNA transfection of quail cells was carried out using the calcium-phosphate method. Focus and colony assays were done as described [36]. QEF were transfected with retroviral expression vectors coding for c-Myc, v-Myc, N-Myc, and L-Myc for the generation of the Myc-transformed cell lines QEF/RCAS-c-Myc, QEF/RCAS-v-Myc, QEF/RCAS-N-Myc, and QEF/RCAS-L-Myc.

### DNA constructs

The *R*Luc-PCA PKA reporter consisting of PKA subunits (RII, PKAc) fused to *R*luc fragment 1 (RII-F[1]) and *R*luc fragment 2 (PKAc-F[2], RII-F[2]) has been described previously [20]. PKAc and RII were replaced with the coding regions of Myc and Max leading to the pcDNA3.1 (backbone vector) *R*luc-PCA expression constructs Myc-F[1]/F[2], Myc(332-439)-F[1]/F[2] and Max-F[1]/F[2] [18]. In detail, PCR-amplified C-terminal (aa 332 to 439) or full length (aa 1 to 439) coding regions of human cellular Myc (specified as Myc or c-Myc; template: c-Myc cDNA from HL-60 cells) and full length chicken Max (aa 1 to 160; template: Max cDNA from CEF) [13] were cloned into the 5′ end of the humanized *R*luc fragments F[1] (aa 1-110) and F[2] (aa 111-310) upstream of the 10-aa linker (GGGGS)_2_. The Venus YFP-based PCA reporter has been described previously [20, 25]. To generate Myc and Max Venus-YFP PCA expression vectors, the coding regions of *R*Luc F[1] and F[2] in Myc-F[1], Myc(332-439)-F[1], Max-F[1] and Max-F[2] were replaced by the Venus-YFP PCA fragments (VenF[1], aa 1-158; VenF[2], aa 159-239). Coding sequences of chicken c-, N-, and L-Myc (template: CEF cDNA) and v-Myc (template: pRc-Myc) [36] were amplified by PCR and inserted into *R*Luc-PCA vectors using the same cloning strategy. For competition experiments and transformation assays, coding regions of human Mxd1 (template: Mxd1 cDNA from SW480 cells) and chicken c-, v-, N- and L-Myc were subcloned into the indicated transient (pcDNA3.1) or retroviral (pRCAS(A)BP) expression vectors, with or without N-terminal hemagglutinin (HA) epitope tags. Using site-directed mutagenesis, proline was substituted for leucine 397 in pcDNA3.1/v-Myc-F[2] and pRc/HA-v-Myc to generate v-Myc(L397P)-F[2] and HA-v-Myc(L397P). Furthermore, S373E and T400E substitutions in c-Myc-F[2] generated single mutants and the double mutant c-Myc(S373E, T400E)-F[2].

### *Renilla* luciferase PCA

Human HEK293 and SW480 cells, or avian QT6 cells were used for the transfections. The indicated combinations of *R*luc-PCA-based hybrid proteins were transiently overexpressed in a 24-well plate format. After 6-, 24-, or 48-h treatment and/or transfection, the growth medium was removed and the cells resuspended in PBS. Stable SW480 cell lines expressing the indicated *R*luc-PCA pairs were grown in 12-well plates for 24 or 48 h. For *R*luc-PCA signal measurements, suspensions of transfected cells were transferred to 96-well white-walled plates and subjected to bioluminescence analysis using the LMaxTM^II^384 luminometer (Molecular Devices). *R*luc bioluminescence signals were integrated for 10 s following addition of the *R*luc substrate benzyl-coelenterazine (5 μM; Nanolight).

### Venus-YFP PCA

QEF grown on transparent slides (μ-slide 4 well, IBIDI, # 80426) were cotransfected with Venus-YFP PCA expression vectors (pcDNA3.1) coding for PKA RII subunits, or for Myc (full length or truncated) and Max proteins fused to the indicated fragments of the Venus-YFP PCA (VenF[1] or VenF[2]) using the TransFectin reagent. 48 h after transfection, cells were subjected to fluorescence imaging. Fluorescent images were visualized using an Axiovert 200M microscope and Axiovision 4.6 software (Carl Zeiss).

### Antibodies

Monoclonal mouse anti-*R*luc-F[1] (Millipore MAB4410), mouse anti-*R*luc-F[2] (Millipore MAB4400), mouse anti HA-tag (Covance), and mouse anti α-tubulin (Sigma-Aldrich) were used.

### Treatment of human and avian cell lines with Myc inhibitors

The indicated human cell lines were treated with the pyridine-based Myc inhibitors KJ-Pyr-9 and KJ-Pyr-10 [18] or with the commercially available Myc inhibitors 10074-G5 (Calbiochem) and 10058-F4 (Calbiochem) [16, 17, 44]. All inhibitor compounds were dissolved in DMSO (10- or 20-mM stock solutions) and diluted in culture medium to yield the indicated final concentrations. The Myc inhibitors were applied to cells for 6, 24, 48, 72, or 240 h. Applied inhibitor compounds were not replenished over the 6-, 24-, 48- or 72-h time periods. For treatment of avian Q/tM8, QT6, and QEF/MC29 cells with Myc inhibitors, cells were seeded into 12-well dishes (3.75 × 10^5^ cells per well) and incubated overnight at 37°C. The culture medium was replaced by 0.5 ml of medium without serum containing KJ-Pyr-9, KJ-Pyr-10, 10074-G5, or 10058-F4 at the indicated final concentrations, and cells were incubated at 37°C. After 2 h, 0.5 ml of medium containing 2x serum and the final concentration of the inhibitor compounds was added. At the indicated time points, cells were counted and microphotographs were taken. To test the effect of Myc inhibitors on re-transformation of conditionally transformed Q/tM8 cells, cells were seeded as above and first cultivated in the presence of doxycycline (1 μg/ml) for 4 d leading to inhibition of v-Myc expression and reversion of the transformed phenotype. Then, the culture medium was replaced by medium lacking doxycycline (leading to re-expression of v-Myc), but containing the indicated Myc inhibitors. Cells were cultivated for 10 d with medium containing the Myc inhibitor exchanged every 80 h, and analyzed as above. QEF/RCAS-c-Myc, -v-Myc, -N-Myc, and -L-Myc cell lines were seeded in 6-well plates at a density of 3.5 × 10^5^ cells per well. 6 or 24 h post-seeding, Myc inhibitors were applied at the indicated concentrations. Northern analysis for monitoring c-*myc* and v-*myc* expression, or Myc target gene expression, was done as described [36].

### Cell proliferation assay

Cells were grown in 6- or 12-well plates. The Myc inhibitors were added to the cell culture medium at the indicated final concentrations. At the indicated time points, the number of leukemia cells (MOLT-4) were directly quantified. Adherent cells (QEF/RCAS-c-Myc, QEF/RCAS-v-Myc, QEF/RCAS-N-Myc, QEF/RCAS-L-Myc, QEF/MC29, Q/tM8, QT6) were trypsinized for cell counting using a Coulter counter. To assess cell viability after inhibitor treatment, trypan blue staining was performed as described [36].

## SUPPLEMENTARY MATERIAL AND FIGURES


