# Supplementary material for: In vivo quantification and perturbation of Myc-Max interactions and the impact on oncogenic potential
Source: Oncotarget. 2014 Oct 12;5(19):8869–78. doi: 10.18632/oncotarget.2588 (PMC4253403; doi:10.18632/oncotarget.2588)
Supplement: Supplementary file 1 [file oncotarget-05-8869-s001.pdf]

In vivo quantification and perturbation of Myc-Max interactions and the impact on oncogenic potential

Supplementary Material

A

bHLH-LZ

```
ck Max MSDNDDIEVESDEEQPRFQSAADKRAHHNALERKRRDHIKDSFHSLRDSVPSLQGEKASR 60
hu Max ............................................................ 60

ck Max AQILDKATEYIQYMRRKNHTHQQDIDDLKRQNALLEQQVRALEKARSSAQLQANYPAADS 120
hu Max ..........................................................T...SS.N 120

ck Max SLYTNPKGSTISAFDGGSDSSSDSEPDEPQSRKKLRMEAS 160
hu Max .....A................E...E............. 160
```

B

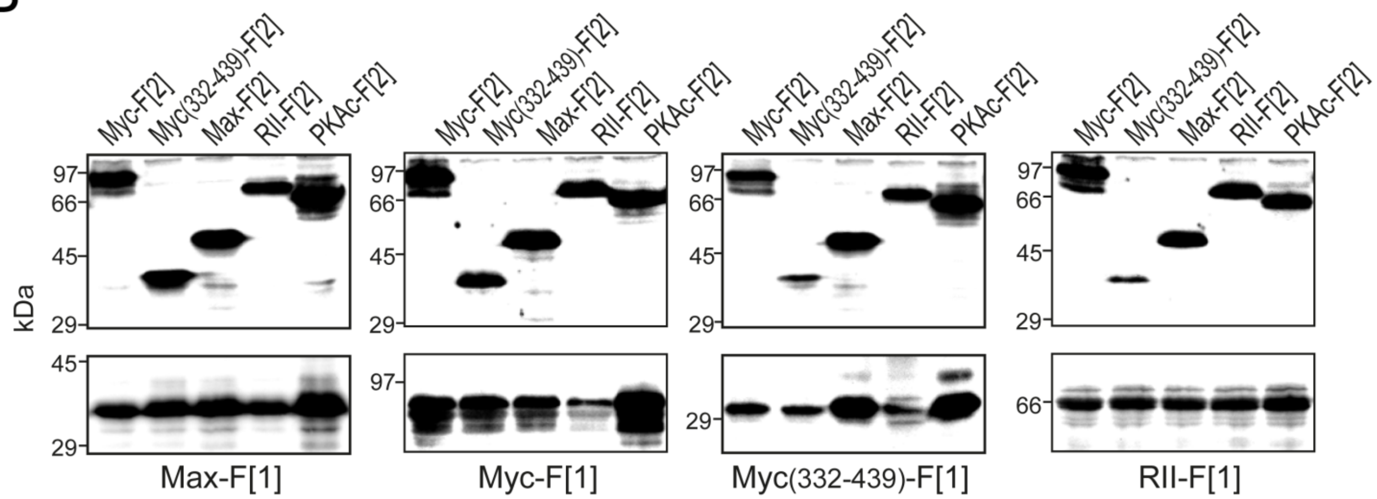

Figure S1: Sequence comparison of human and chicken Max and immunoblot analyses of PCA hybrid proteins. (A) Sequence alignment of chicken (ck) and human (hu) Max proteins (GenBank accession numbers: hu Max, NP_002373; ck Max, P52162) using ClustalW; the bHLH-LZ domain is underlined and shown in blue. **(B)** HEK293 cells expressing the indicated *R*luc-PCA hybrid proteins were subjected to immunoblot analyses with antibodies directed against F[1] or F[2] of the *R*luc-PCA.

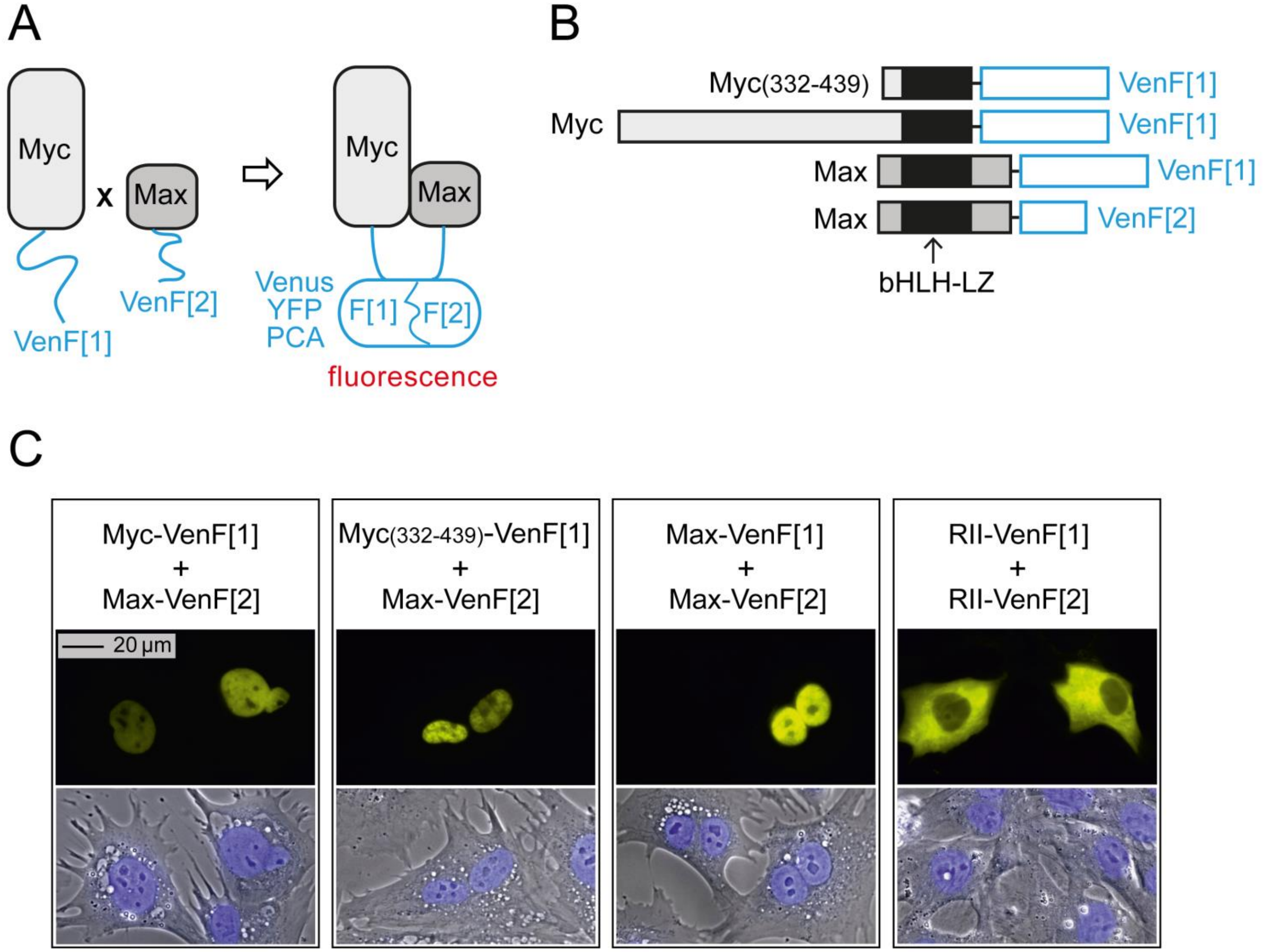

Figure S2: VenusYFP-PCA design and localization of cellular Myc-Max complexes. (A) Schematic depiction of the VenusYFP-PCA based PPI reporter for the *in vivo* localization of protein complexes fused to VenusYFP-PCA fragments 1 (VenF[1]) and 2 (VenF[2]). **(B)** The bHLH-LZ transcription factors Max (full length, aa 1-160) and Myc (full length, aa 1-439; or C-terminal fragment, aa 332-439) were fused at the C terminus to an interjacent 10-aa linker $(GGGGS)_2$ and the VenF[1] and VenF[2] fragments. **(C)** QEF grown on transparent slides were co-transfected with the indicated VenusYFP-PCA expression vectors and subjected to fluorescence imaging. A VenusYFP-PCA based on PKA subunit RII was used as control. Fluorescent images were visualized using an Axiovert200M microscope and Axiovision 4.6 software (Carl Zeiss; representative experiment of n=3). The bottom panels show Hoechst staining of nuclei.

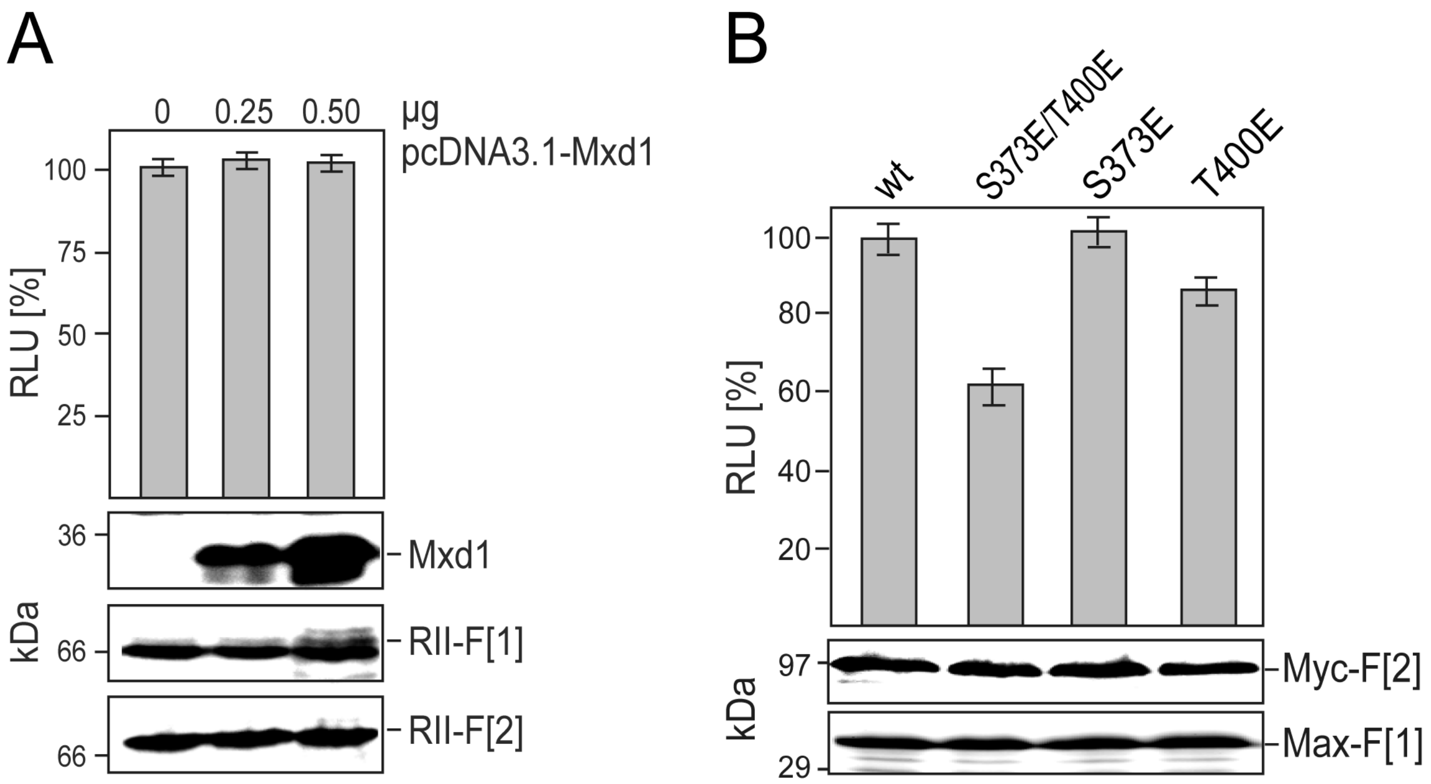

Figure S3: Sensitivity and specificity of the *R*luc-PCA Myc:Max reporter *in vivo*. (A) Control experiment for the analysis shown in Fig. 2D. SW480 cells stably expressing the PKA based *R*Luc-PCA pair RII-F[1]:RII-F[2] were subjected to bioluminescence analysis following transient expression of Mxd1 (HA-tagged). Increasing amounts of pcDNA3.1-Mxd1 vector DNA were transfected (representative of n=3; ± SD from triplicates). Expression of the PCA hybrid proteins and of Mxd1 was analyzed by immunoblotting. **(B)** Analysis of the human c-Myc mutants S373E and T400E. Complex formation between wild type (wt), single, and double mutants of human Myc with Max was determined in a *R*luc-PCA assay of transiently transfected HEK293 cells (representative of n=3; ± SEM of triplicates). The *R*luc-PCA tagged hybrid proteins were analyzed by immunoblotting.

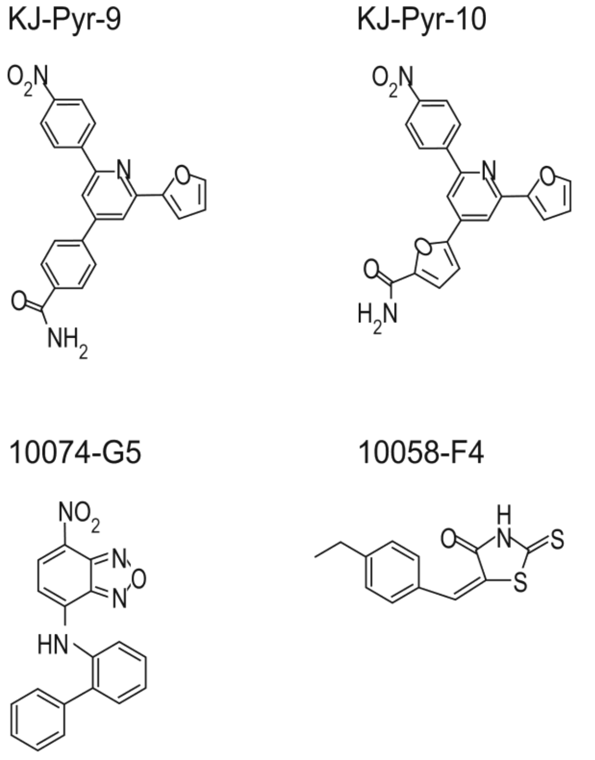

Figure S4: Chemical structures of Myc inhibitor molecules. Shown are the novel pyridine derivatives KJ-Pyr-9 and KJ-Pyr-10 (18), and the structurally unrelated inhibitors Biphenyl-2-yl-(7-nitrobenzo[1,2,5]oxadiazol-4-yl)-amine (10074-G5) and (*Z*,*E*)-5-(4-Ethylbenzylidine)-2-thioxothiazolidin-4-one (10058-F4) (16, 17, 44).

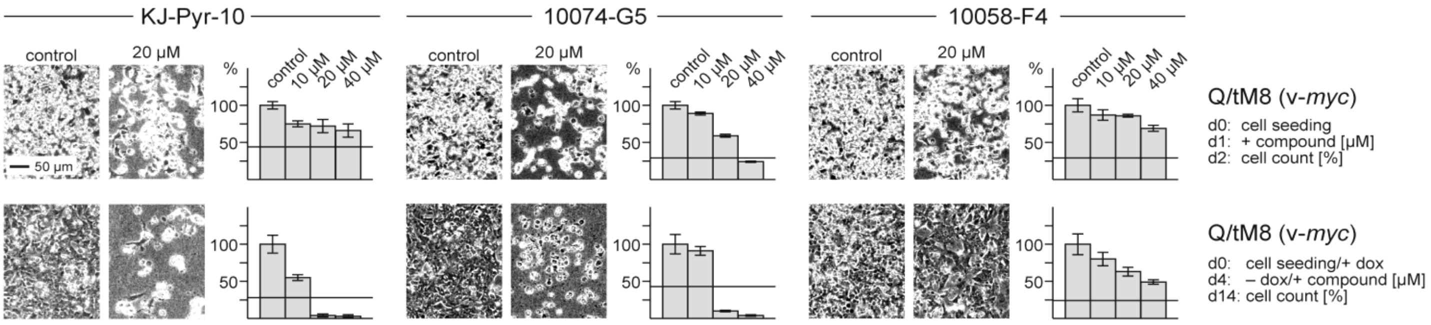

Figure S5: Effect of Myc inhibitors on the proliferation of the tet-off quail cell line Q/tM8, conditionally transformed by the v-*myc* oncogene. *Upper row*: transformed cells grown continuously in the absence of doxycycline were seeded at day zero (d0) onto MP-12 wells (3.75 x 10^5 cells per well). One day after seeding (d1), the inhibitor compounds KJ-Pyr-10, 10074-G5, or 10058-F4 were added at the indicated concentrations, and on the next day (d2) cells were counted and microphotographs were taken. *Bottom row*: cells were seeded at d0 and doxycycline was added to induce reversion of the transformed phenotype. At d4, doxycycline was removed to allow reactivation of the oncogene, and the inhibitor compounds were added simultaneously. At d14, cells were counted and microphotographs were taken. Average numbers of control cells were set to 100%. Horizontal bars indicate the numbers of cells initially seeded.

```
                                                                   MB I
ck c-Myc     MPLSASLPSKNYDYDYDSVQPYFYFEEEEENFYLAAQQRGSELQPPAPSEDIWKKFELLPTPPLSPSRR---------------------   69
MC29 v-Myc   ............................................................M........---------------------   69
ck N-Myc     ..G---MI...P.LEF..L..C..PD.DD--...CGPDSAP-----.G....................AGLQEHPPGGAPLPWGGAALGS   79
ck L-Myc     .-------------ER.AY.H.L.DYDAG----------EDFHRST...........FV.......LGE---------------------   46

                                                                MB II
ck c-Myc     SSLAAASCFPSTADQLEMVTELLGGDMVNQSFICDPDDESFVKSIIIQDCMWSGFSAAAKLEKVVSEKLATYQASRREGGPAAASRPGPP   159
MC29 v-Myc   ............................................................................Q.............   159
ck N-Myc     CRP.DPLDWA.ELLL.PPEA..W.STDGADF.ETGLGASNNLN..............RE...RA.....QS-------KP....PP.P..   162
ck L-Myc     ----RPG.SAAEERGCPPRCC.P---DEPEYL.GTGQLFGNLSAF.LR.........RER...AMT...S--------------------   109

                                           MB IIIa
ck c-Myc     PSGPPPPPAGPAASAGLYLHDLGAAAADCIDPSVVFPYPLSERAPRAAPPGANP--------------AALLGVDTPPTTSSDSEEEQEE   235
MC29 v-Myc   ......................................................--------------......................   235
ck N-Myc     VVPTAAVA.ANSP...PGRAE..GSVPE.V..A....F.VNK.DAAVPSG.ET.RGGRPPRPAGESRASSSS.D..LSDSDDED...EDD   252
ck L-Myc     ---TGT.R.A.HKPSSAPDLGF.SSVSE.V..AA..LC..A.S--------------------------------KV.PS.GSEGQSDS.   164

                MB IIIb                                                  MB IV
ck c-Myc     DEEIDVVTLAEANESE-------SSTESSTEASEEHCKPHHSPLVLKRCHVNIHQHNYAAP-P-----STKVEYPAAKRLKLDSGRVLKQ   312
MC29 v-Myc   ................-------......................................-.-----......................   312
ck N-Myc     E.......VEKRRS.SNKAVTTLTI.VRPKNTTFPSVRTQQNE.I....APIHQ.......S.Y--ME.--EDV.PQ.K..AEVP.PV.P   338
ck L-Myc     G.......VEKRQSLS----------LRKPVTITLRAD.LDPCMKHFHISIHQQ......RS.PDACPQP.SPQQDEEE.LSAAEPPPSH   244

                                                                  bHLH-LZ
ck c-Myc     ISNNR-KCSSPRTSDSEENDKRRTHNVLERQRRNELKLSFFALRDQIPEVANNEKAPKVVILKKATEYVLSIQSDEHRLIAEKEQLRRRR   401
MC29 v-Myc   .....-.......L........................R.............................L.....K...............   401
ck N-Myc     MIQPKS.S....N....DSER..N..I.......D.RS..LT...HV..LVK....A............H.L.AE.QK.LL...K.QA.Q   428
ck L-Myc     ALPDPGLPKAGPG....DVA..KN..Y...K...D.RSR.L.....V.GL.SCP.T......S.SS..LQ.LI.A.R.MA...R...L.Q   334

ck c-Myc     EQLKHKLEQLRNSRA   416
MC29 v-Myc   .....N.........   416
ck N-Myc     Q..LK.I.YK.TC--   441
ck L-Myc     M..LRQIA..KGH--   347
```

Figure S6: Sequence alignment of MC29 v-Myc and chicken Myc family proteins. The sequences of c-Myc, v-Myc, N-Myc, and L-Myc (GenBank accession numbers: ck c-Myc, NP_001026123; MC29 v-Myc, P06295.2; ck N-Myc, chicken genome contig NW_001471673, nt 3920044-3922476; ck L-Myc, XP_425790) were aligned using ClustalW with additional manual adjustments. Residues identical to c-Myc are shown as dots, dashes indicate gaps, conserved Myc boxes (MB) and the bHLH-LZ region are shaded.
